# High AC and DC Electroconductivity of Scalable and Economic Graphite–Diamond Polylactide Nanocomposites

**DOI:** 10.3390/ma14112835

**Published:** 2021-05-26

**Authors:** Jacek Fal, Katarzyna Bulanda, Mariusz Oleksy, Jolanta Sobczak, Jinwen Shi, Maochang Liu, Sławomir Boncel, Gaweł Żyła

**Affiliations:** 1 Department of Experimental Physics, Faculty of Mathematics and Applied Physics, Rzeszów University of Technology, 35-959 Rzeszów, Poland; j.sobczak@prz.edu.pl (J.S.); gzyla@prz.edu.pl (G.Ż.); 2 Department of Polymer Composites, Faculty of Chemistry, Rzeszów University of Technology, 35-959 Rzeszów, Poland; k.bulanda@prz.edu.pl (K.B.); molek@prz.edu.pl (M.O.); 3 International Research Center for Renewable Energy, State Key Laboratory of Multiphase Flow in Power Engineering, Xi’an Jiaotong University, Xi’an 710049, China; jinwen_shi@xjtu.edu.cn (J.S.); maochangliu@mail.xjtu.edu.cn (M.L.); 4 Department of Organic Chemistry, Bioorganic Chemistry and Biotechnology, Silesian University of Technology, 44-100 Gliwice, Poland

**Keywords:** graphite, diamond, polylactide, nanocomposite, electrical properties, electrical conductivity

## Abstract

Two types of graphite/diamond (GD) particles with different ash content was applied to prepare new electroconductive polylactide (PLA)-based nanocomposites. Four samples of nanocomposites for each type of GD particles with mass fraction 0.01, 0.05, 0.10, and 0.15 were prepared via an easily scalable method—melt blending. The samples were subjected to the studies of electrical properties via broadband dielectric spectroscopy. The results indicated up to eight orders of magnitude improvement in the electrical conductivity and electrical permittivity of the most loaded nanocomposites, in reference to the neat PLA. Additionally, the influence of ash content on the electrical conductivity of the nanocomposites revealed that technologically less-demanding fillers, i.e., of higher ash content, were the most beneficial in the light of nanofiller dispersibility and the final properties.

## 1. Introduction

Polylactide (PLA) is an intrinsically electroinsulating, thermoplastic polyester obtained by polycondensation of bio-derived lactic acid [1]. PLA is biodegradable while the overall mechanical characteristics allow its increasing contribution in the pool of everyday life polymers. The PLA or PLA-based materials offer bioresorbable and biosafe solutions for implantology and surgery, 3D-printing filaments, packaging foils, etc. On the other hand, since pristine PLA is brittle, unstable at higher temperature, and flammable, one has observed a remarkably growing interest in improving its physical properties, particularly toward economic and technologically less-demanding solutions. Here, the enhanced electroconductivity is one of the key characteristics [2]. The electrical properties of newly composed materials such as polymer nanocomposites are very important in many areas of real-life their application. Such nanocomposites could be applied in inexpensive sensors [3], electronics [4], and electromagnetic interference (EMI) shielding [5]. It is therefore essential to examine the electrical properties of such new nanocomposite materials, specifically their AC (alternating current) and DC (direct current) electroconductivity.

Among numerous routes of manufacturing PLA nanocomposites, high-shear mixing of melts [6], extrusion [7,8], melt [9] or solution spinning [10,11], and capillary-action [12] can be recalled. Nevertheless, the first listed method remains the most frequently used due to its simplicity and scalability. While from the nanofiller point-of-view, these are the carbon nanomaterials which promise the highest potential in the manufacturing electroconductive composites. These nanomaterials were indeed commonly applied since they might additionally offer a significant enhancement in the mechanical and/or thermal performance [13]. There are several dozens of works revealing substantial enhancement of the above properties of PLA nanocomposites, but the most prospective effects were obtained for yet uneconomic carbons such as graphene or carbon nanotubes or their mixtures with graphite [14], making the target materials rather arduously scalable.

A prospective compromise between the enhancement of thermophysical properties and economy of the composites could be achieved by other carbon-based fillers such as graphite or carbon black, both composed from micro- to nanoparticles. Therefore, graphite-PLA (15 wt.%) nanocomposites of electroconductivity 0.0125 μS/cm were manufactured via high-shear mixing [15]. The authors used PLA with 40,000 g/mol molar mass and natural spheroidal graphite of an average diameter ca. 500 μm. Accordingly, Żenkiewicz et al. prepared graphite-PLA nanocomposite (up to 50 wt.% of graphite) but based on PLA with 70,000 g/mol and graphite particles of diameter less than 44 μm (85 wt.%). The nanocomposites displayed electrical conductivity of 1.67 × 10^−5^ μS/cm [16]. The electrical properties of graphite-PLA nanocomposites were also studied by other researchers, but they used various modified nanofillers such as exfoliated graphite [15], graphite oxide [17], or polymer functionalized graphite [18] to obtain enhanced electrical and mechanical properties, in reference to the neat PLA matrix.

Here, we demonstrate, that conveniently synthesized (commercially available), and hence readily accessible graphite–diamond (GD) nanoparticles could serve as nanofillers toward PLA nanocomposites of high, yet unrecorded AC- and DC-electroconductivity, which is unique as compared to the previously reported results. The GD nanoparticles emerged indeed as fully compatible with the PLA matrix enabling scalable melt processing toward the fully functional materials.

## 2. Materials and Methods

Two types of GD nanoparticles of different ash contents—produced by controlled dry detonation synthesis followed by purification procedures—were purchased from PlasmaChem GmbH (Berlin, Germany) and were labelled as GD6 and GD03—for 6 wt.% and 0.3 wt.% ash content, respectively. Both samples contained min 20 wt.% of diamond nanoparticles of the average primary particle size of 4 nm while the second component was graphite, typically micron-size of an angular shape of the flake. The key properties of GD nanoparticles as well as their morphological and chemical characterization, including scanning electron microscopy (SEM), energy-dispersive X-ray spectroscopy (EDS), and X-ray diffraction (XRD) analysis were presented elsewhere [19]. As the matrix, pristine/neat and colorless PLA (Propox, Chwaszczyno, Poland) in the form of 3D-printing filament (head temperature recommended for 3D-printing (463.15–478.15 K (190–205 °C)) mixed with the maleic anhydride grafted polyethylene (F) (Fusabond E226, DuPont, Wilmington, DE, USA) and anhydrous glycerin (G) (Chempur, Piekary Śląskie, Poland) as a compatibilizer and plasticizer were used, respectively.

**Figure 1:**
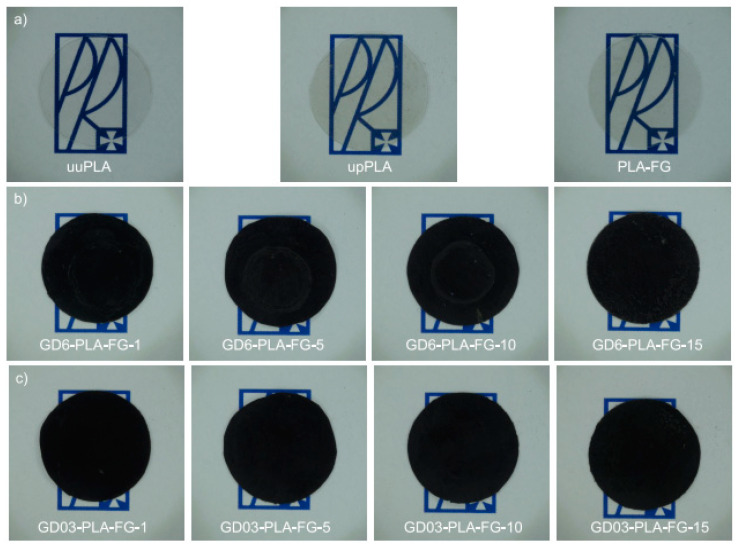
The pictures of (**a**) PLA; (**b**) graphite/diamond with 6 wt.% ash content PLA nanocomposites, and (**c**) graphite/diamond with 0.3 wt.% ash content PLA nanocomposites of various nanofiller contents. uuPLA—unfilled and unprocessed PLA; upPLA—unfilled and processed PLA; PLA-FG—PLA with plasticizer and compatibilizer; GD6—graphite/diamond mixture with 6 wt.% ash content; GD03—graphite/diamond mixture with 0.3 wt% ash content; numbers—wt.% of filler (GD6, GD03).

The GD/PLA nanocomposite and the reference PLA samples were prepared via high-shear mixing in the following steps. Firstly, the appropriate amounts of individual components were weighted using an analytical balance (Pioneer Semi-Micro PX225DM, OHAUS Corporation, Parsippany, NJ, USA) and mixed. Subsequently, the pre-mixtures were placed in a co-rotating twin-screw extruder (HAAKE MiniLab II, Thermo Fisher Scientific, Karlsruche, Germany) to obtain the corresponding nanocomposite thread. The extrusion was carried out using a 50 rpm-screw speed at 463.15 K. The threads were granulated and used for the injection molding HAAKE MinJet II (Thermo Fisher Scientific, Karlsruche, Germany) to obtain bar-shaped samples (10 *×* 60 *×* 1 mm^3^). By using a press and cylindrical knife, the samples were formed into discs of 20 mm in diameter (Figure 1). The rationale behind the selection of the filler content range (0–15 wt.%) was based on the efficiency-to-economy ratio—in terms of the electrical performance, and tangible deterioration of mechanical performance at the higher contents. We used therefore the 15 wt.% value as the top-line matching the literature review and hence enabling further comparative studies. Importantly from the manufacturing point-of-view, we have not encountered any significant problems in manufacturing nanocomposites as compared to the manufacturing of the neat PLA. While the nanocomposites emerged as slightly more flexible upon bending than the neat PLA samples.

The samples were labelled in a following way: uuPLA (unfilled and unprocessed PLA—the sample was subjected only to the injection process), upPLA (unfilled and processed PLA—the sample was first melted in a co-rotating twin-screw extruder then subjected to the injection process), PLA-FG (mixture of PLA, Fusabond and glycerine), and FILLER-PLA-FG-x (where: FILLER is GD6 or GD03, x is wt.% of filler). As visible by an unarmed eye, independently from the nanofiller content, all of the PLA nanocomposites emerged as practically non-transparent and black, opposite to the transparent neat PLA samples of the above, various manufacturing history. 

The fracture surface of the nanocomposites was observed with SEM (Hitachi S- 3400N (Hitachi Ltd., Tokyo, Japan). The high- and low-vacuum mode (LV-50 Pa) with a backscattered electron detector (BSE) and a 5 kV-accelerating voltage was applied. 

The electrical properties of GD/PLA nanocomposites were investigated with Concept 80 System (Novocontrol GmbH, Montabaur, Germany) coupled with a temperature control unit (Quatro Cryosystem, Novocontrol GmbH, Montabaur, Germany). All measurements were conducted in the temperature range of 298.15–333.15 K with a frequency starting from 0.1 to 10^6^ Hz with a logarithmic scale in 55 steps. More details on the measurement procedure and information about standard deviation of measurements (<6%) are available elsewhere [19,20,21].

## 3. Results and Discussion

**Figure 2:**
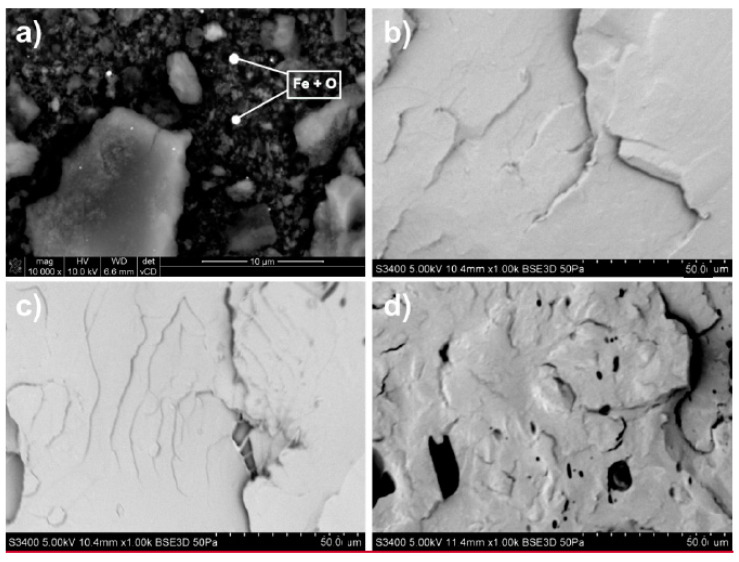
SEM images of: (**a**) GD nanoparticles of 6 wt.% ash content; magnification 10,000×, and fractured surface of: (**b**) uuPLA, (**c**) upPLA, and (**d**) PLA-FG; magnification 1000×. uuPLA—unfilled and unprocessed PLA; upPLA—unfilled and processed PLA; PLA-FG—PLA with plasticizer and compatibilizer.

Morphology of GD nanoparticles (Figure 2a) and various neat PLA materials (Figure 2b–d) was analyzed using SEM. 

GD6 nanoparticles emerged as up to few-micron, angular graphite microparticles surrounded by numerous tiny, few-nanometer size nanodiamonds. Importantly, EDS analysis revealed that the main components of the ash, visible as the brighter spots, were iron and oxygen [22]. The role of Fe_x_O_y_ ash nanoparticles, in the light of the dispersion of electroactive nanoparticles, and hence electrical properties of the final composites, cannot be underestimated.

**Figure 3:**
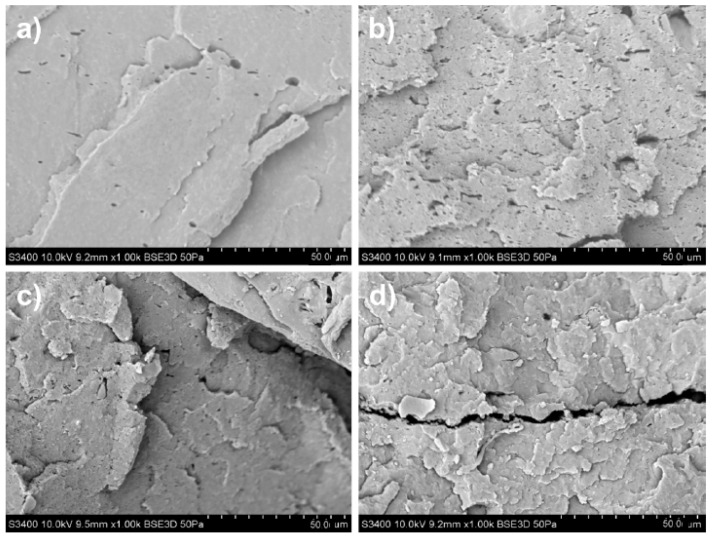
SEM images of fractured surface morphology for (**a**) GD6-FG-PLA-1, (**b**) GD6-FG-PLA-5, (**c**) GD6-FG-PLA-10, (**d**) GD6-FG-PLA-15, Magnification 1000×, PLA-FG—PLA with plasticizer and compatibilizer, GD6—graphite/diamond mixture with 6 wt.% ash content, numbers—wt.% of filler (GD6).

**Figure 4:**
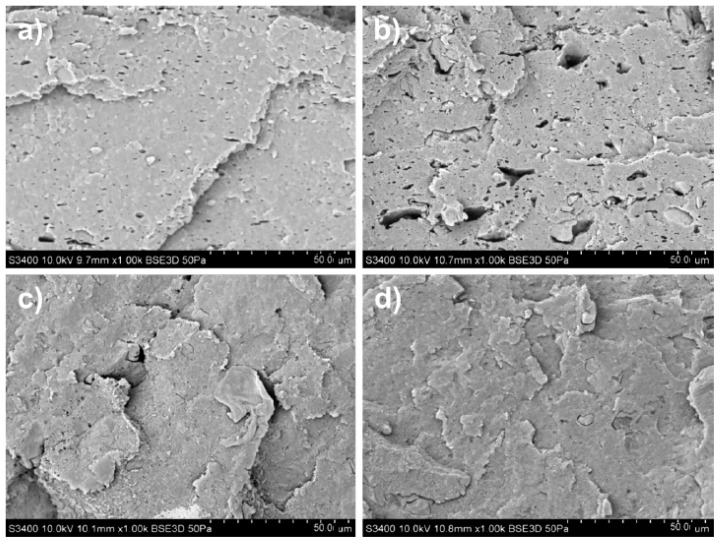
SEM images of fractured surface morphology for (**a**) GD03-FG-PLA-1, (**b**) GD03-FGPLA-5, (**c**) GD03-FG-PLA-10, (**d**) GD03-FG-PLA-15. Magnification 1000×; PLA-FG—PLA with plasticizer and compatibilizer, GD6—graphite/diamond mixture with 0.3 wt% ash content; numbers—wt.% of filler (GD03).

As for the nanocomposites, GD6-PLA (Figure 3) and GD03-PLA (Figure 4) exhibited high dispersibility, and therefore homogeneity of GD nanoparticles in the matrix—there are indeed no visible agglomerates of nanoparticles on the surface. 

The GD6-PLA-FG-1 nanocomposite was characterized, at the fracture surface, with smooth zones of cracks and irregular faults between planes of splitting. With the increase of the nanofiller content, an increase in the number of the planes was observed with a simultaneous decrease in their size. Additionally, in the case of GD6-PLA-FG-5 nanocomposite, numerous voids on the crack surface could be captured. Their manifestation could be associated with the application of plasticizer and compatibilizer. In turn, the GD03-PLA-FG nanocomposites were characterized mainly by morphology of the brittle crack surface. GD03-PLA-FG-1 nanocomposite, similar to GD6-PLA-FG-1, was found as revealing larger zones of cracks with distinct faults between the parallel crack planes. The size of such zones decreased with the nanofiller content in the matrix, while their edges become more uneven.

**Figure 5:**
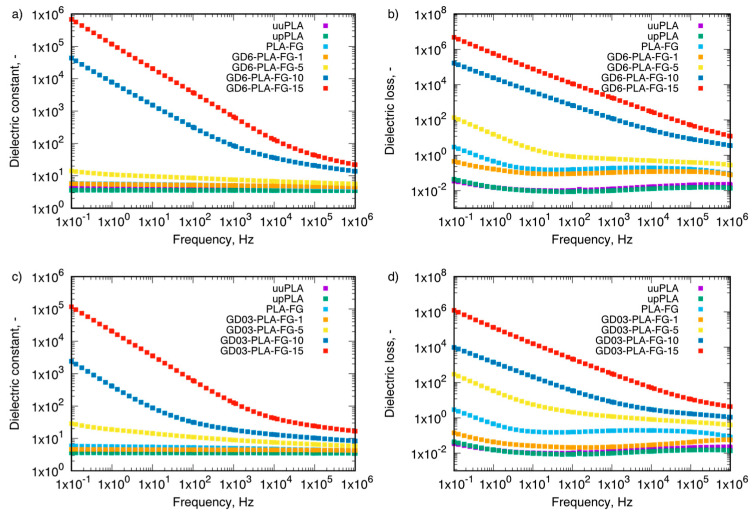
Dielectric constant of: (**a**) GD6-PLA-FG nanocompoites, (**c**) GD03-PLA-FG nanocomposite and dielectric loss (**b**) GD6-PLA-FG nanocomposites, (**d**) GD03-PLA-FG nanocomposites at 298.15 K. uuPLA—unfilled and unprocessed PLA, upPLA—unfilled and processed PLA, PLA-FG—PLA with plasticizer and compatibilizer, GD6—graphite/diamond mixture with 6 wt.% ash content, GD03—graphite/diamond mixture with 0.3 wt% ash content, numbers—wt.% of filler (GD6, GD03).

Coming to the electrical properties of GD-PLA nanocomposites, both parts of permittivity (dielectric constant and dielectric loss) of the two types of GD/PLA nanocomposites were investigated (Figure 5). 

The obtained results indicated that addition of the nanofillers caused an increase in dielectric constant as well as dielectric loss for the nanocomposites, in the whole examined frequency range. Additionally, a decrease in the frequency entailed an increase in both dielectric constant and loss which could be immediately assigned to the formation of 3D-conduction paths, and hence the increase in the proportion of electrical conductivity in the nanocomposites. Moreover, as the dominating nanoparticle phase is graphite, though stabilized by diamond nanoparticles, the most probable conduction mechanism emerges as the formation of 3D conduction paths as shown previously [23]. The exceptions were the neat PLA samples for which the influence of frequency on the dielectric constant was negligible. Wang et al. [24] hypothesized that an increase in the nanofiller content in the matrix could reduce the average distance between the nanoparticles, hence enabling the more efficient electron tunneling. This explanation falls within the phenomena describing formation of the percolation thresholds. Additionally, in the low-frequency range, there is a possibility of occurrence Maxwell/Wagner/Sillars effect related to the charge blocking on the internal phase boundaries. This effect manifests itself in the strong increase in dielectric constant with a decrease in frequency, which can be observed especially for 10 and 15 wt.% filler content—for both types of the tested GD-PLA nanocomposites. Additionally, the dielectric behavior of pristine PLA can be characterized by α- and β-relaxation processes occurring in the low and high frequencies, respectively, as presented by Badia et al. [25]. Nevertheless, α-relaxation is typically activated at higher temperatures, and, in fact, it has not been observed in our study. On the other hand, β-process could be also observable even at the temperature higher than 398.15 K, but here, incorporation of the GD nanoparticles into the PLA matrix led to its clear disappearance with the filler content at the frequency range from 0.1 to 1 MHz.

**Figure 6:**
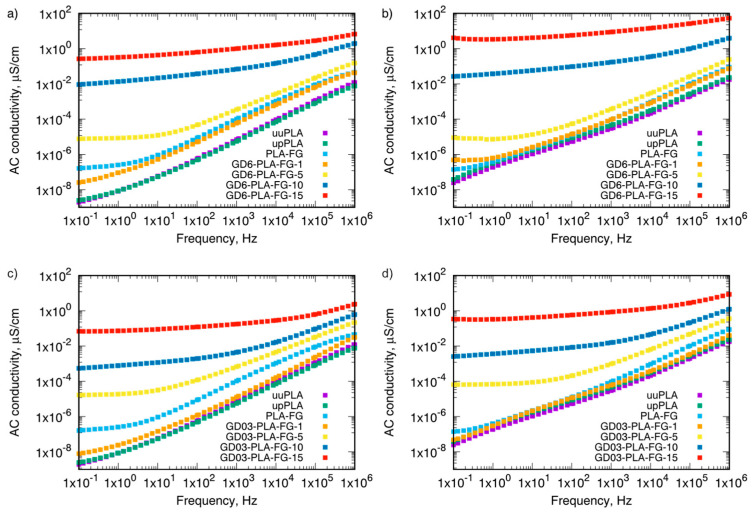
AC conductivity of (**a**) GD6-PLA-FG nanocomposites, and (**c**) GD03-PLA-FG nanocomposites at 298.15 K; (**b**) GD6-PLA-FG nanocomposites, and (**d**) GD03-PLA-FG nanocomposite at 333.15 K. uuPLA—unfilled and unprocessed PLA, upPLA—unfilled and processed PLA, PLA-FG—PLA with plasticizer and compatibilizer, GD6—graphite/diamonod mixture with 6 wt.% ash content, GD03—graphite/diamond mixture with 0.3 wt% ash content, numbers—wt.% of filler (GD6, GD03).

Figure 6 presents the AC conductivity dependence on frequency for two types GD/PLA nanocomposites with nanofiller concentration from 0.00 to 0.15 wt.%, at 298.15 and 333.15 K.

The results showed that the injection process had only a minor effect on the electrical conductivity of PLA in the absence of GD nanofiller (both unprocessed and processed). Indeed, the processed PLA exhibited slightly higher conductivity than the unprocessed which could be attributed to the minimal increase in the decomposition- and hydrolysis-derived lactic acid content. This, in turn, means that, in the presence of matrix water, lactic acid dissociates to lactate and hydronium hence slightly increasing the overall electrical conductivity via the ionic mechanism. On the other hand, the addition of compatibilizer and plasticizer increased electrical conductivity in the whole tested frequency range. As PLA-FG (0.0) sample contained maleic anhydride grafted polyethylene, upon partial hydrolysis under processing and conditioning at ambient humidity, the higher value of conductivity in comparison to the uuPLA (0.0) sample can be referred to the presence of transformation of maleic anhydride into the maleic acid-like moieties. Hence, in total, the increase in the concentration of hydronium cations. There are also visible strong dependences of AC conductivity on the frequency for the neat PLA samples in the low frequency range, which is typical for electroinsulating materials [26]. In turn, the filled PLA showed a significant frequency-dependence only for their higher filler loadings. In the low frequency range, below approximately 20 Hz, the AC conductivity remained almost unaffected by the frequency changes. The addition of GD nanoparticles to the PLA matrix caused an increase in the AC electrical conductivity in the whole tested frequency range, particularly at the low frequency, where this increase was the most striking. Only for the lowest nanoparticle content, a decrease in the AC conductivity was observed—as compared to PLA with compatibilizer and plasticizer for both types of GD nanoparticles.

**Figure 7:**
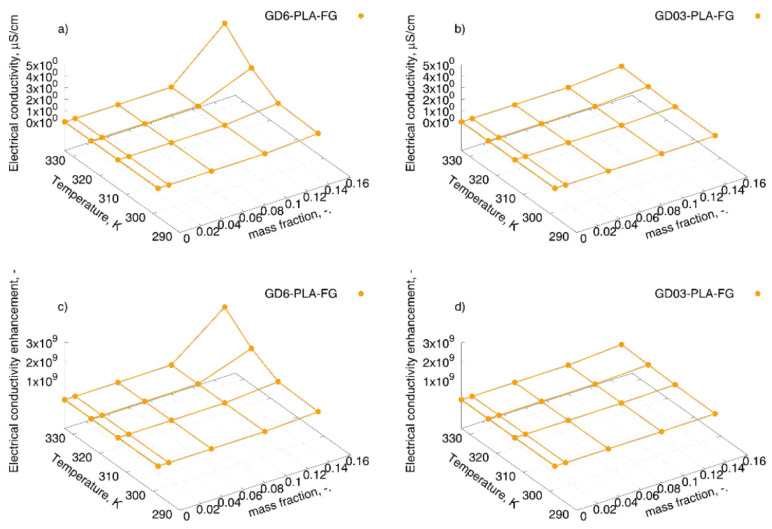
Electrical conductivity of: (**a**) GD6-PLA-FG, and (**b**) GD03-PLA-FG nanocomposites, and electrical conductivity enhancement designated as the ratio of conductivity at 0.1 Hz of the specimen to the conductivity of uuPLA in each temperature for (**c**) GD6-PLA-FG, and (**d**) D03-PLA-FG.

Based on the region insensitive on frequency, DC conductivity was designated as the value of AC conductivity at the lowest tested frequencies (0.1 Hz) [27], and presented as the electrical conductivity enhancement in Figure 7 and Table 1.

The analysis of the data presented shows a little impact of the addition of filler with a mass fraction up to 15 wt.% below which electrical conductivity enhancement was visible but much less than in the case of nanocomposites with 15 wt.% in PLA-FG matrix. There, an increase of eight and seven order of magnitudes for GD6/PLA and GD03/PLA nanocomposites was observed, respectively. Such a significant increase in the electrical conductivity could be caused by the formation of conducting 3D-paths through the GD nanoparticles with the addition of stabilizing ash nanoparticles. Moreover, the temperature effect is noticeable, and an increase in electrical conductivity with temperature was observed. This effect could be assigned to the intrinsic behavior of electrical conductivity of graphite which increases with rise of temperature. Hence, these are the graphite particles which would play the dominating role in the conductivity mechanism [28]. 

**Figure 8:**
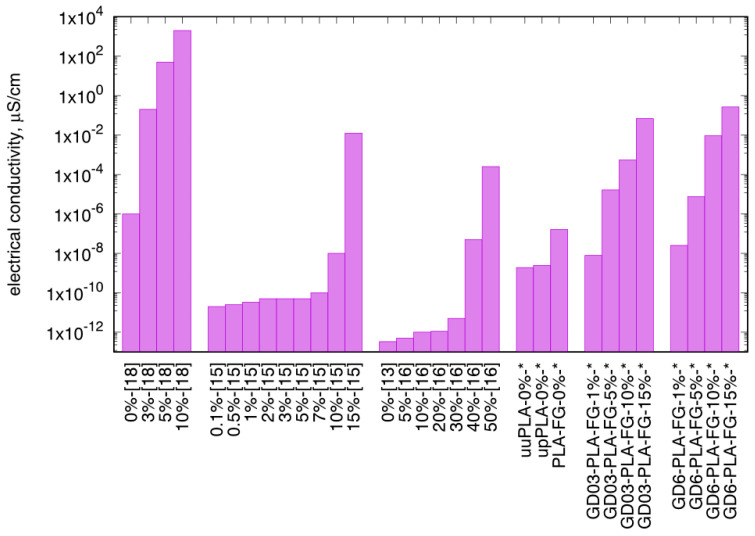
Comparison of electrical conductivity of graphite-polylactide nanocomposites obtained by various researchers with different techniques; *—this study at 298.15 K.

The obtained results compared to those available in the literature—for nanocomposites prepared with just pristine graphite as a filler in PLA matrix—show electrical conductivity higher at least one order of magnitude than presented by other researchers, as shown in Figure 8. Similarly, studies on PLA and graphite as matrix and filler, respectively, was presented by Kim et al. [15], where the maximum electrical conductivity was achieved—as in our case—for 15 wt.% filler content in PLA, but the outcome in the electrical conductivity itself was found as 95% lower than in our case. What is more, nanocomposites with the extremely high graphite filler loading (up to 50 wt.%) prepared by Żenkiewicz et al. [16] showed lower electrical conductivity than that presented in this study. The only exception is the *functionalized graphene* composite prepared by Cheng et al. [18] which was manufactured via a difficult to scale-up casting method.

Referring back to the fracture surfaces of GD nanoparticles (Figure 2a) and PLA nanocomposites (Figure 3 and Figure 4), the comparison of electrical conductivity recorded for both types of GD/PLA nanocomposites indicates that the major impact on electrical conductivity enhancement was constituted by the ash content. Indeed, it was the only differentiating factor among the nanoparticles, most probably and primarily due to the stabilizing effect on the 3D-network of electroconductive nanofillers.

## 4. Conclusions

The two types of new PLA-based nanocomposites with commercially available GD nanoparticles (of different ash contents) as fillers were prepared via an easily scalable melt blending method. The carbon GD nanoparticles were all well-dispersible in the PLA matrix by means of compatibilizer and plasticizer, and ready-to-use without any additional modifications.

Importantly, the most electroconductive PLA nanocomposites revealed an eight order of magnitudes enhancement in the electrical conductivity without any nanoparticle modification such as surface functionalization. We believe this is a suitable starting point for the development of highly electroconductive PLA nanocomposites readily applicable in EMI shielding, sensors, or 3D printing electronics. Last but not least, the PLA nanocomposites were characterized by increasing electrical conductivity with the filler loadings for both types of GD nanoparticles. But, for the PLA nanocomposites based on GD of higher ash content (6 wt.%), one order of magnitude higher electrical conductivity than that for GD-PLA with a lower ash loading (0.3 wt.%) was found. This fact demonstrates the actual synergy as the stage of purification in the synthesis of GD nanoparticles can be neglected. Finally, as the PLA matrix is biodegradable and, contrarily, GD are thermodynamically stable, recyclability of the filler appears as the straightforward and convenient route.

## Figures and Tables

**Table 1:** Electrical conductivities and their enhancements designated as the ratio of conductivity at 0.1 Hz of the specimen to the conductivity of uuPLA in each temperature for two types of the graphite/diamond mixtures PLA nanocomposites.

Sample Type	Mass Fraction, φ_m_	Electrical Conductivity, µS/cm			
	**–**	298.15 K	313.15 K	323.15 K	333.15 K
unfilled PLA	uuPLA (0.0)	1.91 × 10^−9^	3.22 × 10^−9^	6.31 × 10^−9^	2.43 × 10^−8^
	upPLA (0.0)	2.45 × 10^−9^	3.74 × 10^−9^	8.75 × 10^−9^	3.84 × 10^−8^
	PLA-FG (0.0)	1.62 × 10^−7^	9.66 × 10^−8^	6.39 × 10^−8^	1.39 × 10^−7^
GD6-PLA-FG	0.01	2.53 × 10^−8^	5.62 × 10^−8^	9.00 × 10^−8^	4.83 × 10^−7^
	0.05	7.59 × 10^−6^	6.89 × 10^−6^	2.51 × 10^−6^	8.89 × 10^−6^
	0.10	9.21 × 10^−3^	1.26 × 10^−2^	1.85 × 10^−2^	2.64 × 10^−2^
	0.15	2.65 × 10^−1^	4.24 × 10^−1^	1.85 × 10^0^	4.09 × 10^0^
GD03-PLA-FG	0.01	7.83 × 10^-9^	2.05 × 10^−8^	1.24 × 10^−7^	4.74 × 10^−8^
	0.05	1.65 × 10^-5^	2.90 × 10^−5^	3.09 × 10^−5^	6.47 × 10^−5^
	0.10	5.43 × 10^-4^	1.18 × 10^−3^	1.70 × 10^−3^	2.54 × 10^−3^
	0.15	6.85 × 10^-2^	1.19 × 10^−1^	2.15 × 10^−1^	3.41 × 10^−1^
	**Mass Fraction,** **φ_m_**	**Electrical Conductivity Enhancement, –**			
	–	298.15 K	313.15 K	323.15 K	333.15 K
unfilled PLA	uuPLA (0.0)	1.00 × 10^0^	1.00 × 10^0^	1.00 × 10^0^	1.00 × 10^0^
	upPLA (0.0)	1.28 × 10^0^	1.16 × 10^0^	1.39 × 10^0^	1.58 × 10^0^
	PLA-FG (0.0)	8.50 × 10^1^	3.00 × 10^1^	1.01 × 10^1^	5.72 × 10^0^
GD6-PLA-FG	0.01	1.33 × 10^1^	1.75 × 10^1^	1.43 × 10^1^	1.99 × 10^1^
	0.05	3.98 × 10^3^	2.14 × 10^3^	3.98 × 10^2^	3.66 × 10^2^
	0.10	4.83 × 10^6^	3.91 × 10^6^	2.93 × 10^6^	1.09 × 10^6^
	0.15	1.39 × 10^8^	1.32 × 10^8^	2.92 × 10^8^	1.68 × 10^8^
GD03-PLA-FG	0.01	4.11 × 10^0^	6.37 × 10^0^	1.97 × 10^1^	1.95 × 10^0^
	0.05	8.66 × 10^3^	9.01 × 10^3^	4.89 × 10^3^	2.67 × 10^3^
	0.10	2.85 × 10^5^	3.66 × 10^5^	2.70 × 10^5^	1.05 × 10^5^
	0.15	3.59 × 10^7^	3.71 × 10^7^	3.41 × 10^7^	1.40 × 10^7^

uuPLA—unfilled and unprocessed PLA, upPLA—unfilled and processed PLA, PLA-FG—PLA with plasticizer and compatibilizer, GD6—graphite/diamond mixture with 6 wt.% ash content, GD03—graphite/diamond mixture with 0.3 wt% ash content, numbers—wt.% of filler (GD6, GD03).

## Data Availability

The data presented in this study are available on request from the corresponding author.
